# Non-Additive Transcriptional Profiles Underlie Dikaryotic Superiority in *Pleurotus ostreatus* Laccase Activity

**DOI:** 10.1371/journal.pone.0073282

**Published:** 2013-09-05

**Authors:** Raúl Castanera, Alejandra Omarini, Francisco Santoyo, Gúmer Pérez, Antonio G. Pisabarro, Lucía Ramírez

**Affiliations:** Genetics and Microbiology Research Group, Department of Agrarian Production, Public University of Navarre, Pamplona, Navarre, Spain; University of California Riverside, United States of America

## Abstract

**Background:**

The basidiomycete *Pleurotus ostreatus* is an efficient producer of laccases, a group of enzymes appreciated for their use in multiple industrial processes. The aim of this study was to reveal the molecular basis of the superiority of laccase production by dikaryotic strains compared to their parental monokaryons.

**Methodology/Principal Findings:**

We bred and studied a set of dikaryotic strains starting from a meiotic population of monokaryons. We then completely characterised the laccase allelic composition, the laccase gene expression and activity profiles in the dikaryotic strain N001, in two of its meiotic full-sib monokaryons and in the dikaryon formed from their mating.

**Conclusions/Significance:**

Our results suggested that the dikaryotic superiority observed in laccase activity was due to non-additive transcriptional increases in *lacc6* and *lacc10* genes. Furthermore, the expression of these genes was divergent in glucose- *vs.* lignocellulose-supplemented media and was highly correlated to the detected extracellular laccase activity. Moreover, the expression profile of *lacc2* in the dikaryotic strains was affected by its allelic composition, indicating a putative single locus heterozygous advantage.

## Introduction

**Figure 1:**
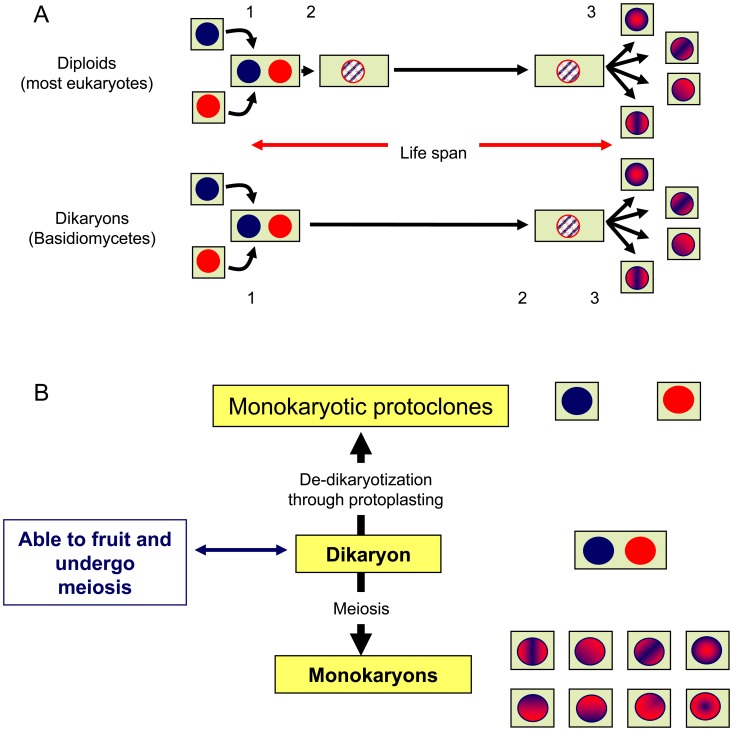
Diploid *versus* dikaryotic life cycle. (A) In most diploid organisms, plasmogamy (1) is immediately followed by karyogamy (2), and the cells contain the two parental genomes fused in the same nucleus throughout the life cycle until meiosis (3) occurs. In dikaryons, however, karyogamy occurs at the end of the life cycle immediately before the onset of meiosis. In these cells, the two parental nuclei remain unfused, sharing the same cytoplasm throughout the life cycle. (B) The dikaryotic condition permits not only the generation of monokaryotic (haploid) offspring through meiosis but also to recover the two parental nuclei through protoplasting. All of these types of nuclei are able to establish new monokaryotic mycelia (cell lines).

In most higher organisms, karyogamy occurs shortly after plasmogamy, and the two equivalent genomic copies present in the cell are fused into a single diploid nucleus. In contrast, in basidiomycetes, the two genomic copies are kept separated in the two parental nuclei, but share the same cytoplasm throughout most of the life cycle. This condition is known as dikaryotic and terminates when karyogamy occurs immediately prior to the onset of the meiotic divisions that produce the haploid monokaryotic spores (Figure 1A). In dikaryons (n+n) and in diploids (2n), the total genetic complement is similar, but its organisation makes them behave differently. Somatic recombination in dikaryons creates new allelic combinations, which produce new gene expression patterns and phenotypic traits, whereas meiosis in diploids reshuffles allelic combinations, but does not create new allelic combinations. Furthermore, the presence and spacing of the two different nuclei in the same cytoplasm create new internuclear gene cross-talk regulatory networks that permit dikaryons to better adapt to their different environmental conditions. The differences in gene expression under monokaryotic and dikaryotic conditions can be analysed in detail because it is possible to separate the two nuclei in the dikaryon by protoplasting, which produces two independent monokaryotic mycelia (each mycelia having only one of the nuclei present in the dikaryon) (Figure 1B). Thus, the genetic effect contributed by each of the two nuclei can be individually studied in dikaryotic and monokaryotic conditions, which make dikaryons a good model system to study gene expression at the intra- and intergenomic levels.

In maize plants Shull [1] defined heterosis as the outperformance of the hybrid offspring of two inbred lines compared to the performance of the parental lines. In classical genetic terms, it is the result of dominance or overdominance effects. Dominance stresses the complementation of deleterious alleles homozygous in the parental inbred lines by superior alleles in the hybrids, and overdominance postulates that different alleles in the hybrid (genetic heterozygosity) could be responsible for a superior performance (heterosis) of the hybrids. In the case of fungi, the use of the concepts of dominance and overdominance, as they have been used in plants, in studies of gene expression in monokaryons and dikaryons could be controversial because fungal gene transcript levels in hybrids sometimes exhibit an additive pattern resulting from the contribution of each allele in the hybrid, whereas a large body of literature describes a non-additive pattern of gene expression in plants [2].

White rot fungi are able to degrade the wood polymer lignin as part of the process of gaining access to cellulose that will be used as a carbon source. Ligninolysis occurs by oxidative attacks on different lignin components carried out by secreted enzymes from the groups of laccases (Lac, EC 1.10.3.2), manganese peroxidases (MnP, EC 1.11.1.13), lignin peroxidases (LiP, EC 1.11.1.14), and versatile peroxidases (VP; EC 1.11.1.16) [3]. The white rot basidiomycete *Pleurotus ostreatus* (Jacq.: Fr) Kumm. has been studied as a model organism for basidiomycete genetics and genomic studies because it is one of the more important industrially produced edible mushrooms and is a source for industrially produced ligninolytic enzymes. In addition, it participates in biotechnological industrial applications and processes, such as biopulping [4], bioremediation [5], [6] and enzymatic pretreatment of lignocellulose for the production of biofuels. *P. ostreatus* is representative of one of two alternative lignin-degrading strategies because it produces laccase (Lac), versatile peroxidase (VP) and the so-called dye-decolorizing peroxidase (DyP), but not lignin peroxidases (LiP), in contrast to the strategy followed by the other model organism *Phanerochaete chrysosporium*, which produces LiP, but any of the other enzymes mentioned above (although both produce manganese peroxidises, MnP ) [7], [8].

*P. ostreatus* grows in its natural environment on tree stumps, grows in industry on cereal straw-based substrates, and can be easily cultured in the laboratory and brought to fruiting *in vitro* [9]. *P. ostreatus* monokaryons and dikaryons are both able to grow on the aforementioned substrates and degrade lignin, although differences have been observed that correspond to the monokaryotic or dikaryotic nature of the mycelium. In the process of lignocellulose degradation, three main areas may be studied: growth rate, enzyme production and gene expression, all of which contribute to the efficiency of the ligninolytic process. Thus, the dikaryotic nature of *P. ostreatus* offers the opportunity to study the genetic and genomic contributions of the monokaryotic/dikaryotic condition of the mycelium in each of these areas. This opportunity is reinforced by the availability of the sequence of these two genomes, which are present in a natural dikaryotic strain (strain N001), and by a set of previous genetic studies that have determined the genotype of monokaryons and dikaryons derived from *P. ostreatus* strain N001.

In this study, we have examined the effect of the monokaryotic/dikaryotic condition on growth rate, Lac activity production and Lac gene expression (*lacc* genes). We found that (i) the three parameters behaved non-additively in dikaryons compared to their parental monokaryons, (ii) monokaryons and dikaryons displayed different laccase activity profiles that could be explained by non-additive increases in the expression of *lacc2*, *lacc6* and *lacc10*; and (iii) dikaryons with high extracellular Lac activity in glucose-based media exhibited low activity in lignocellulose-based media and vice-versa, which correlated with the divergent expression of *lacc6*, *lacc2*, and *lacc10* in both type of cultures. Taken together, these *lacc* genes may be classified into three clusters/groups according to their expression profiles in lignocellulose-based media.

## Materials and Methods

### Fungal Strains and Culture Conditions

Seventeen strains of *Pleurotus ostreatus* were used in this study: (i) the dikaryotic strain N001, from which the two parental protoclones PC9 and PC15 were obtained by de-dikaryotisation [10], [11]; (ii) 12 full-sib (individuals sharing both parents) monokaryons (mk01, mk02, mk06, mk20, mk36, mk37, mk61, mk63, mk67, mk69, mk88, mk93), which were selected from among the offspring used to construct the genetic linkage map of the strain N001 [12], and (iii) four dikaryons (67×69, 61×63, 93×69, 36×69), which resulted from the mating of the corresponding compatible monokaryons.

Fermentation in Glucose Supplemented Cultures (GSC) was carried out in 135 ml of M7GY medium containing (per litre) 2 g ammonium tartrate, 0.5 g MgSO_4_·7H_2_O, 1 g KH_2_PO_4_, 0.5 g KCl, 10 g glucose, 1 g yeast extract and 1 ml trace elements solution [0.1 g Na_2_B_4_O_7_·H_2_O, 0.07 g ZnSO_4_, 0.01 g CuSO_4_·5H_2_O, 0.01 g MnSO_4_·4H_2_O, 0.05 g FeSO_4_·7H_2_O, and 0.01 g (NH_4_)_6_Mo_7_O_2_·4H_2_O per litre]. For RNA-seq experiments, SMY medium (10 g of sucrose, 10 g of malt extract and 4 g of yeast extract per litre) was used. Fermentation in Lignocellulose Supplemented Cultures (LSC) was carried out on 10 g of milled wheat straw (particle size <4 mm) soaked with 120 ml of distilled water in glass flasks at a surface to volume ratio of 0.368 cm^−1^. Inocula of the GSC and LSC cultures consisted of 15 ml of homogenised active growing mycelia cultured in liquid M7GY. Solid cultures were carried out on malt extract agar (MEA, 20 g malt extract and 15 g agar per litre) supplemented with Remazol Brilliant Blue R (RBBR, MEA-R) at a final concentration of 0.2 g/l. Media sterilisation was performed at 121°C for 20 min. The LSC cultures were incubated in static conditions at 25°C in the dark. The GSC cultures were incubated in an orbital shaker (150 rpm) at 25°C in the dark.

### Linear Growth Rate

Linear growth rates were determined in three biological replicates in MEA-R. A 4-mm^2^ piece of actively growing mycelium was placed in the centre of the Petri dish, and the fungal growth was measured as the radial increase in the colony (mm) per day. Four orthogonal measures were made per plate daily.

### Decolourisation Assay

RBBR decolourisation was assessed in the MEA-R by visual inspection via an evaluation of the disappearance of the blue colour from the plate. A range from I to IV (Figure S1 in File S2) was used to evaluate the decolourizing activity of each strain on the basis of the size and intensity of the decolourisation halo.

### Laccase Activity Assay

Lac activities were determined spectrophotometrically using 2,6-dimethoxyphenol (DMP) as substrate, by measuring the appearance of the dimeric product resultant of DMP oxidation (ε _468_ = 49,600 M^−1^cm^−1^) after incubation for 60 s at 25°C. For these incubations, 90 to 450 µl of the sample was added to 500 µl of a 10 mM DMP solution in 0.1 M sodium acetate buffer (pH 5.0) up to 1 ml final volume with the same buffer. The enzyme activities were expressed in international units (U), where one unit of enzyme activity is defined as the amount of enzyme that oxidised 1 µmol of substrate in 1 min.

### Nucleic Acid Extraction and Reverse Transcription

The mycelia were harvested, frozen and ground in a sterile mortar in the presence of liquid nitrogen. Total RNA was extracted from approximately 200 mg of deep frozen tissue using Fungal RNA E.Z.N.A Kit (Omega Bio-Tek, Norcross, GA). The integrity of the total RNA was determined by denaturing electrophoresis on 1% (w/v) agarose gels. Nucleic acid concentrations were measured in duplicate using a Nanodrop™ 2000 spectrophotometer (Thermo-Scientific, Wilmington, DE), and the purity of the total RNA was estimated by the 260/280 nm absorbance ratio. Samples were DNAse treated using 1 U of RQ1 DNase (Promega, Madison, WI) per µg of RNA according to the manufacturer instructions and then purified and concentrated using RNeasyR MinieluteR Cleanup (Quiagen Iberia S.L., Madrid, Spain). One microgram of total RNA was reverse-transcribed into cDNA in a 20 µl volume using the iScript cDNA Synthesis kit (BIO-RAD Laboratories S.A., Alcobendas, Spain) according to the manufacturer’s instructions.

### Primer Design and Verification

The primers described by Castanera *et al.* [13] were used for the amplification of *lacc* transcripts (Table 1). Primers corresponding to the reference gene panel were designed for the filtered model transcripts sequences of PC9 and PC15 (http://www.jgi.doe.gov/) using the PrimerQuest^SM^ tool (Integrated DNA Technologies, Madrid, Spain). Primer specificity was validated *in silico*, by performing Primer-Blast against all of the model transcripts of both the monokaryotic protoclones. The absence of nonspecific PCR artefacts was confirmed by melting-curve analysis.

**Table 1:** PCR primers, amplicon length, and amplification efficiency of the laccase and reference genes.

	Transcript ID					
Gene	PC15 v2.0^a^	PC9 v1.0^b^		Primer sequence	Amplicon length (bp)	Amplificationefficiency
*lacc1*	1043420	90578	Fw	GGTACATCCTAGCACCCAATG	80	1.79
			Rv	GACGAGATCAGTTTCCAAGAGG		
*lacc2*	1067328	116143	Fw	CCCTGGCAGATTGGTATCATG	142	1.80
			Rv	ATGACAGCGTAAGGGACAAG		
*lacc3*	1102751	123288	Fw	TCGTTTCCGTCTCGTTTCTC	134	1.69
			Rv	CTGCGAAGATTTGGATGCTG		
*lacc4*	1077328	65894	Fw	CCCCATCCTTTCCATCTTCAC	72	1.78
			Rv	GTAGTTATACACCGAGCTTCCG		
*lacc5*	1094975	90812	Fw	CGC ATT TGC CGC TTT CTT	136	1.77
			Rv	GGTGACTAGGACTGAGTATCTC		
*lacc6*	1113032	81104	Fw	GTACAACTACGAAAACCCCG	140	1.71
			Rv	CAAGGTCAAGATGCCAGT		
*lacc7*	1077468	60400	Fw	GTTGATAGCCTCCAGATCTTCG	142	1.76
			Rv	GTAGGATGGCGGAGTTGATG		
*lacc8* c	1106925		Fw	CATTGGCTGTGACTCGAA	137	1.73
			Rv	GGATCAGAGAATAGCGTTGG		
*lacc9*	1089733	81107	Fw	CTATCCTTCGGTATGCTGGTG	145	1.77
			Rv	ATATTGATGTCTGCGCCTCC		
*lacc10*	1089723	81117	Fw	CCTACTTCCCCTTTGGCTATC	122	1.76
			Rv	ATGACGAGCAAAGAGTGACC		
*lacc11*	1043488	90573	Fw	CCTGAATGGTCTGATCTCTGC	93	1.74
			Rv	CCTATGACTTGGGCTCTTCG		
*lacc12*	1094965	90834	Fw	GTACTCATTTTCGGCTCCTG	84	1.79
			Rv	CCACGTAGTCCATCGCAATA		
*cyc*	1035989	86026	Fw	ACCTGGCGTCGTTATCTCAAGTGT	122	1.81
			Rv	TTGGCCGAACAGCGAATGGTTTAC		
*actin2*	1114037	90915	Fw	GCCGTGATCTTACCGACTA	134	1.55
			Rv	CTCCTGCTCAAAGTCCAA		
*cyt-c*	1113744	112752	Fw	GCCTCATAAAGTCGGTCCTAAC	127	1.79
			Rv	CTCAAATAGGGTGTTCTCGTCC		
*lip*	48183	75430	Fw	AGGTTTGGCGGGATACAATACGGA	132	1.79
			Rv	ATGCAAGCATCATTTGCGCCGAAC		
*pep*	1092697	115017	Fw	TGATTCCAGAGGACAAGGACGCAA	148	1.79
			Rv	AAATCTTCCGCGATACGGGTCACT		
*phos*	49987	49690	Fw	CATCGCAAATCATCGATCGCACCA	125	1.81
			Rv	GCTCTCCAGCCAATGCACCAATTT		

a,b Transcript identification numbers correspond to the PC15 version 2.0 and PC9 version 1.0 genomes available at www.jgi.doe.gov.

c Only present in the PC15 genome.

### Allele-type Identification

A combination of *in silico* and experimental procedures was used to determine the genotype of the *lacc* genes (PC9 or PC15 type) present in the monokaryotic and dikaryotic strains. The origin of eight of the 12 alleles in mk61 and mk63 was determined by analysing the segregation of linked molecular markers that were previously used to construct the N001 genetic map [12]. For mk61 and mk69, the resolution of the genetic map of N001 was too low to enable the determination of the allelic type of *lacc1*, *lacc4*, *lacc6* and *lacc11*. Thus, RT-PCR was performed using the cDNA template and primers listed in Table S1 in File S1. The PCR products were purified, sequenced and aligned, and the allelic types were identified by SNP inspection.

### Real-time Quantitative PCR

RT-qPCR experiments were performed using a Bio-Rad CFX96 thermal cycler. SYBR green fluorescent dye was used to detect product amplification. Each reaction was set to a final volume of 20 µl and contained 1X IQ SYBR green Supermix (Bio-Rad Laboratories S.A., Alcobendas, Spain), 100 nM forward and reverse primers, and 1 µl of 1∶100 dilution of RT product in nuclease-free water. The amplification program was performed according to the following protocol: 5 min at 95°C followed by 40 cycles of 15 s at 95°C and 30 s at 65°C and a final melting curve with increments of 0.5°C every 5 s in a linear gradient from 65°C to 95°C. Baseline correction and Crossing Point (Cp) acquisition were performed using the Bio-Rad CFXManager. The reactions were performed in triplicate in 96-well microtiter plates, and no-template controls were included for each master mix (one for each primer set). The reaction efficiencies were sample-estimated from a Window-of-Linearity set in the exponential phase of the fluorescence history, which was plotted in log scale using the LinReg tool [14].

### Selection and Validation of Reference Genes

A reference panel of 6 candidate genes for RT-qPCR data normalisation was selected by analysing RNA-seq data of the N001 strain cultured in three different media and harvested at different growth stages (GSC in SMY, LSC on wheat straw and LSC on poplar wood chips, data not shown). The reference candidate genes were selected on the basis of their highly similar expression in the three different media. RPKM (reads per kilobase per million mapped reads) values were used to estimate transcript abundance, and Z-test statistics were used to detect genes that were differentially expressed. Four genes displaying highly stable expression in the three conditions (p<0.05) as well as two common housekeeping genes (actin and cytochrome-c) were selected as the reference gene panel. GeNorm [15] and NormFinder [16] algorithms were applied to verify the expression stability of the six candidates under our experimental conditions using the strain N001. The ideal number of reference genes was calculated, and a reference index consisting of the best-performing candidates was used for RT-qPCR data normalisation.

### Quantification Strategy and qPCR Data Normalisation

Relative expression analyses were performed to estimate the differential amount of transcripts between the samples, and two approaches were used to achieve this purpose: (i) relative quantities (RQ), calculated in arbitrary units according to equation 1, were derived from the comparative ΔCp method described in the Applied Biosystems User Bulletin No. 2 (P/N 4303859), and (ii) gene expression ratios were calculated according to equation 2 [17], which is expressed in the logarithmic scale (fold change, FC).
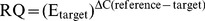
(1)

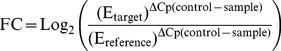
(2)


In these equations, target and reference are the gene of interest and the reference gene index (geometrical mean of reference genes), respectively, and E_target_ and E_reference_ are the corresponding amplification efficiencies. The control is the reference condition (GSC at day 0), and the sample is the tested condition.

### Multivariate Analysis

Hierarchical clustering was performed using the Genex software (MultiD Analyses AB, http://www.multid.se). FC data sets were autoscaled to classify the genes according to their expression profiles without taking into account the amplitude of the changes [18], [19]. Heatmaps were constructed using the Ward’s algorithm [20].

Principal Component Analysis (PCA) and Self Organising Map (SOM) analyses were performed using mean centered data to account for the magnitude of changes in the classification of the samples.

### Statistical Analyses

SPSS for Windows V8.0.1S software (SPSS Inc., Chicago, IL) was used to perform the normality tests (Kolmogorov-Smirnov) and analyses of variance (ANOVA and Tukey’s Post-hoc test). The REST random pairwise reallocation test [21] was used to reveal differences between the gene expression data sets.

### Experimental Procedure

Twelve full-sib monokaryons ferrying chromosome VIII of the PC9 type were selected among the 80 progeny of strain N001. A Quantitative Trait Locus (QTL, R^2^ = 20.27) involved in the growth rate control and a Lac gene (*lacc2*) mapped to this chromosome. Six of the 12 monokaryons (mk36, mk61, mk63, mk67, mk69, mk93) with good performance for growth rate and RBBR decolourisation were selected as putative parental dikaryons. Four dikaryons (67×69, 61×63, 93×69, and 36×69) were obtained by mating compatible monokaryons as previously described. The growth rates and extracellular laccase activities were analysed in the dikaryons and in their respective parental monokaryons. Considering the enzymatic profile displayed by the four dikaryons in GSC and LSC, the dikaryotic strain 61×63 was selected for further analysis. Finally, the dikaryons N001 and 61×63 and the monokaryons mk61 and mk63 were used to analyse the time-course transcription of the entire *lacc* multigene family.

## Results

### Genetic Control of Growth Rate in Monokaryons and Dikaryons

To study the effect of the monokaryotic and dikaryotic condition on the growth rate, the 17 *P. ostreatus* strains that were described in the Materials and Methods section were used. The genotypes of N001 and the monokaryons used in this study were previously known [12]. The two genomes present in N001 (those of protoclones PC9 and PC15) have been independently sequenced, and the genotype of their corresponding chromosomes is known. All of the monokaryotic strains used in this study were selected for having the chromosome VIII variant present in the PC9 protoclone.

The growth rate of the monokaryons varied from 2.30 to 3.57 mm/day, and the strains were grouped into four growth rate categories (Table 2) (Groups I to IV, p<0.05). The growth rate of the dikaryotic strain N001 (4.03 mm/day) was slightly faster compared to the fastest monokaryon, although when it was introduced into the statistical analysis, it was grouped with the three fastest growers, which formed a fifth category. Simultaneously, four decolourizing qualitative levels could be differentiated (Table 2, Figure S1 in File S2). In general, the faster the monokaryons grew, the higher their RBBR-decolourizing activity. This study selected six monokaryons (mk36, mk61, mk63, mk67, mk69, and mk93) on the basis of their growth rates and decolourizing abilities to generate four new dikaryotic strains 36×69, 61×63, 67×69, and 93×69. These four dikaryons displayed growth rates that ranged from 4.23 to 4.94 mm/day, which were significantly higher compared to their parental monokaryons and the N001 strain, but lower than what it would be expected under the assumption of an additive model (Table 3).

**Table 2:** Growth rate^a^ and RBBR decolorization by *P. ostreatus* strains.

Strain	I^b^	II^b^	III^b^	IV^b^	V^b^	VI^b^	VII^b^	VIII^b^	Decolorization
mk20	2.30±0.15								I
mk88	2.48±0.18	2.48±0.18							II
mk01	2.53±0.08	2.53±0.08							I
mk02	2.63±0.08	2.63±0.08	2.63±0.08						nd^c^
mk61		2.97±0.18	2.97±0.18	2.97±0.18					III
mk67		3.02±0.34	3.02±0.34	3.02±0.34					III
mk06			3.21±0.12	3.21±0.12					II
mk37			3.21±0.16	3.21±0.16					III
mk93			3.23±0.30	3.23±0.30					II
mk63				3.43±0.30	3.43±0.30				III
mk36				3.57±0.20	3.57±0.20				III
mk69				3.57±0.13	3.57±0.13				IV
N001					4.03±0.15	4.03±0.15			–d
36×69						4.23±0.14	4.23±0.14		–d
61×63							4.84±0.23	4.84±0.23	–d
67×69								4.89±0.15	–d
93×69								4.94±0.08	–d
Signf. e	0.83	0.12	0.08	0.08	0.10	1.00	0.08	1.00	

a Growth rate measured in mm/day.

b Groups defined using ANOVA and Tukey *post hoc* test.

c nd = no decolorization.

d =  not measured.

e Signf. = Significance level.

**Table 3:** Genetic effect in growth rate.

				Column 1	Column 2	Column 3	Deviations		
Monokaryons^a^				Additive model^b^	New dikaryon	N001 model	Columns 2–1	Columns 3–1	Columns 2–3
mk61	2.97	mk63	3.43	6.4	4.84	4.03	−1.56	−2.37	0.81
mk36	3.57	mk69	3.57	7.14	4.23	4.03	−2.91	−3.11	0.2
mk93	3.23	mk69	3.57	6.8	4.94	4.03	−1.86	−2.77	0.91
mk67	3.02	mk69	3.57	6.59	4.89	4.03	−1.7	−2.56	0.86

a Growth rate in mm/day.

b This model considers the sum of the two monokaryotic strains.

### Genetic Control of the Extracellular Lac Activity in GSC and LSC Cultures

**Figure 2:**
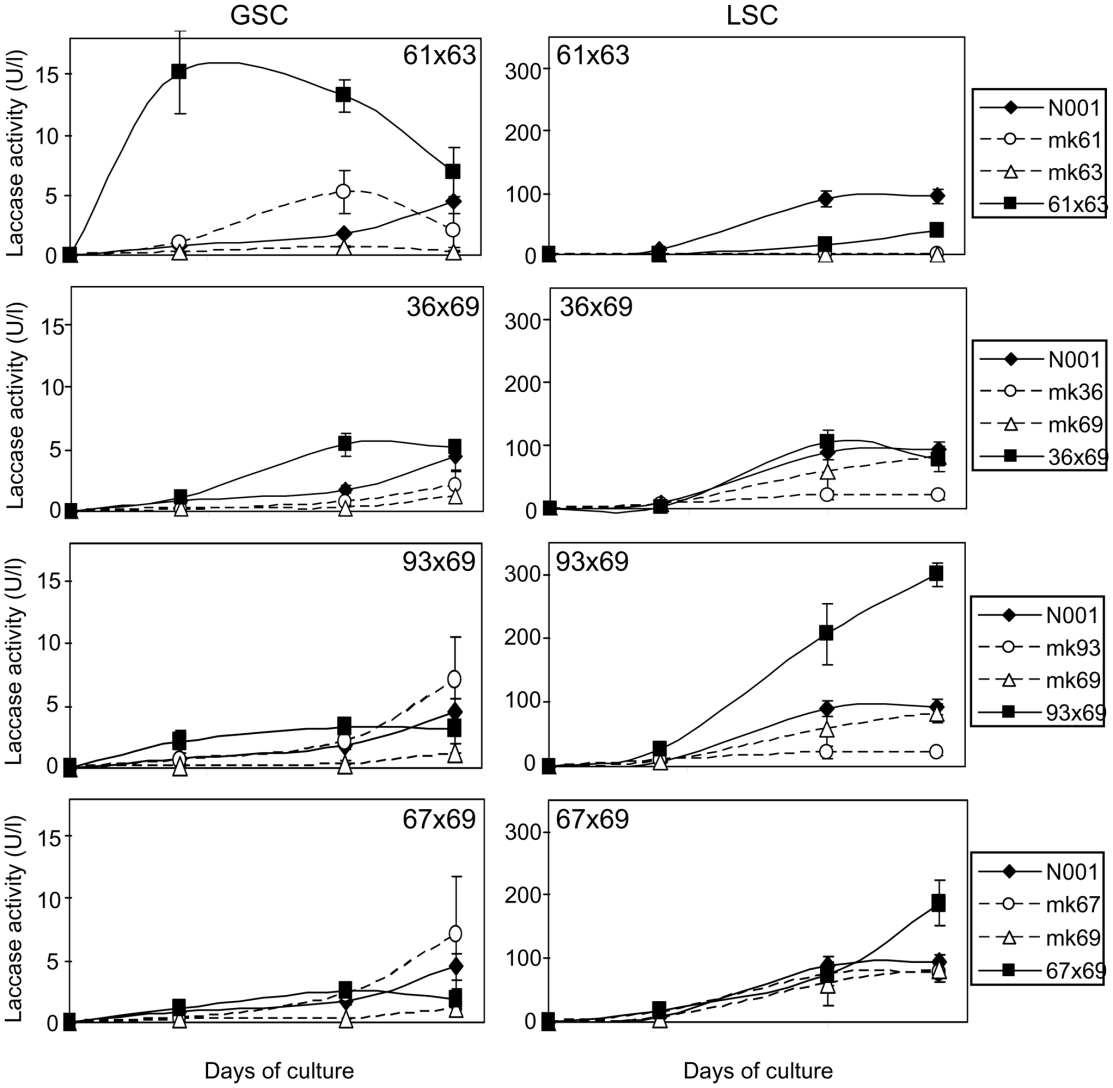
Time course of laccase activity. The evolution of the secreted laccase activity during GSC and LSC cultures of the dikaryons 61×63, 36×69, 93×69, 67×69, their corresponding monokaryotic parental strains (mk61, mk63, mk36, mk69, mk93 and mk67), and the model dikaryotic strain N001. The values represented the mean of three biological repetitions. Standard deviations are presented in Table S2 in File S1.

To study the contribution of the monokaryotic/dikaryotic condition on the laccase activity secreted into the medium during GSC or LSC fermentation, we analysed Lac activity in the parental monokaryons mk36, mk61, mk63, mk67, mk69, and mk93 and in the four derived dikaryons (36×69, 61×63, 67×69, and 93×69) during the culture cycle (Figure 2). In the GSC cultures, the Lac activity observed in the dikaryons was similar to that of their respective monokaryotic parental strains (p<0.05, Table S3 in File S1), in all cases but 61×63. The highest released laccase activity was observed in the cultures of this dikaryon (13.23±1.33 U/l). Two different Lac activity time profiles were observed: the dikaryotic strains 61×63 and 36×69 showed activities that were higher than those displayed by their corresponding monokaryotic parentals, whereas the dikaryons 93×69 and 67×69 showed lower activity values. Table 4 shows the Lac activity values of the new dikaryotic strains compared to those obtained under the assumption of an additive model and the strain N001. We observed a clear and high overdominance effect in laccase activity only in strain 61×63 (deviation of 8.64 U/l) when it is grown in GSC, while the behaviour of strain N001 was similar to that observed in the three new dikaryons.

**Table 4:** Genetic effect in enzymatic activities.

					Column 1	Column 2	Column 3	Deviations		
Condition	Monokaryons				Additive model^a^	New dikaryon	N001 model	Columns 2–1	Columns 3–1	Columns 2–3
**GSC**	mk61	2.75	mk63	0.39	3.14	11.78α	2.34	8.64	−0.80	9.43
	mk36	0.98	mk69	0.57	1.54	3.89β	2.34	2.35	0.80	1.54
	mk93	3.28	mk69	0.57	3.84	2.86	2.34	−0.99	−1.50	0.51
	mk67	3.23	mk69	0.57	3.80	1.86	2.34	−1.94	−1.46	−0.49
**LSC**	mk61	0.12	mk63	0.50	0.62	17.67β	63.98	17.05	63.37	−46.32
	mk36	16.36	mk69	49.24	65.60	62.37	63.98	−3.22	−1.61	−1.61
	mk93	18.04	mk69	49.24	67.28	178.41^αβ^	63.98	111.13	−3.30	114.43
	mk67	54.79	mk69	49.24	104.03	93.73^αβ^	63.98	−10.30	−40.05	29.75

a This model considers the sum of the two monokaryotic strains.

α Means that the enzymatic activity of this dikaryon is significantly different to both of its monokaryotic counterparts (p<0.05).

β Means that the enzymatic activity of dikaryon N001 is significantly different to this new dikaryon (p<0.05).

In LSC cultures, there were two prominent observations: (i) the Lac activity secreted into the culture medium was much higher than in the GSC cultures in all strains but mk61 and mk63. Thus, a clear overdominance effect was observed in the dikaryotic strains, 93×69 and 61×63 (Table 4), which showed mean Lac activities of 178.4 and 17.7 U/l, respectively, whereas an additive effect was observed in strains 36×69 and 67×69. (ii) The genetic effect was divergent from that observed in the GSC cultures (Figure 2) due to the dikaryons that performed the best in GSC (61×63 and 36×69) were low performers in LSC. In contrast, those that showed low laccase activity in GSC (93×69 and 67×69) were the best performers in the LSC cultures.

In the LSC cultures, the mk61 and mk63 strains showed nearly no Lac activity (ranging from 0.1 to 0.5 U/l) whereas the corresponding dikaryon (61×63) showed rather poor activity whose time profile markedly diverged from that observed in the GSC cultures (Figure 2). The differences in the Lac activities observed in the monokaryons and dikaryons, and the divergent behaviour of strain 61×63 in the two culture conditions tested motivated us to study the variation of the expression of the laccase genes in GSC and LSC in the monokaryotic and dikaryotic conditions.

### Identification and Validation of Reference Genes for qPCR Analysis

**Figure 3:**
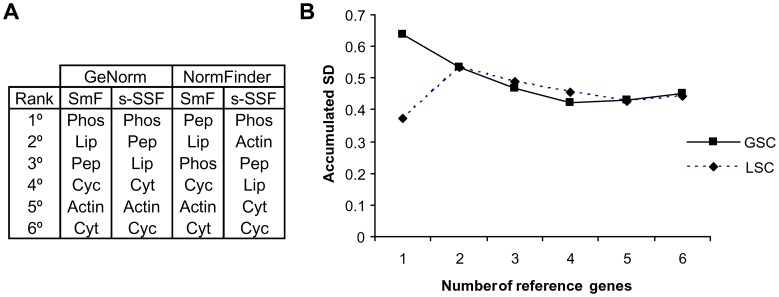
Selection and validation of the reference genes for qPCR analysis. The gene expression stability ranking was obtained by GeNorm and NormFinder (A), and NormFinder accumulated standard deviations for the increasing number of reference genes (B). The reference genes, their gene ID in the *P. ostreatus* PC15 v2.0 genome assembly (http://genome.jgi-psf.org/PleosPC15_2/PleosPC15_2.home.html), and their functional annotation were: *phos* (49987, purine phosphorylase), *lip* (1052421, lipase), *pep* (1092697, peptidase S9), *cyc* (1035989, cyclin-like F-box), a*ctin2* (1114037, actin/actin-like), and *cyt-c* (1113744, cytochrome c).

To determine the appropriate reference genes for quantitative PCR (qPCR) analysis of the expression of the genes of the laccase family, the expression stability of six candidate reference genes (Table 1) was assayed in seven samples of strain N001 at culture days 0, 4, 10 and 14 in GSC, and days 4, 10 and 14 in LSC. The GeNorm algorithm identified *phos* as the most stable gene in GSC and LSC, while NormFinder ranked *pep* and *phos* as the best reference genes in GSC and LSC, respectively (Figure 3A). The accumulated Normfinder standard deviations of increasing number of reference genes were analysed (Figure 3B) to determine the best number of reference genes to be used. As a consequence of this analysis, a group of three genes formed by *pep, phos* and *lip* was selected for data normalisation.

### Transcriptional Analysis of the *lacc* Gene Family in GSC and LSC Cultures

Strain 61×63 was the only strain where the occurrence of enzyme activities could be assigned in the LSC cultures to the dikaryotic nature of the mycelia because no enzymatic activity was detected in the parental monokaryons. In contrast, it was detected in the dikaryon (Figure 2). To study the effect of the monokaryotic *vs.* dikaryotic condition on laccase gene expression in different culture media, we analysed the transcription of the members of this gene family using RT-qPCR in the dikaryotic strain in two steps: (i) transcriptional analysis using the RQ approach and (ii) gene expression profiling using the FC approach and multivariate analysis.

#### (i) Transcriptional analysis using the RQ approach

The transcription of each gene was calculated in comparison to a reference index (consisting of three experimentally validated reference genes), and expressed in relative quantities (RQ, Materials and Methods equation 1). Due to the stability of the reference index between the media and strains (Figure 3, Figure S2 in File S2), the relative quantities of the four strains appeared in the same arbitrary units, and thus could be compared quantitatively to study the different types of gene action (Tables 5 and 6).

**Table 5:** Genetic effect in GSC laccase transcription^a^.

			Column 1	Column 2	Column 3	Deviation (2–1)		Deviation (3–1)		Deviation (2–3)	
Gene	mk61	mk63	Additive model^b^	61×63	N001 model	Units	%	Units	%	Units	%
*lacc1*	0.17	0.08	0.25	0.09	0.02^αβ^	−0.16	−63	−0.22	−90	0.07	273
*lacc2*	0.00	0.00	0.00	0.00	0.08^αβ^	0.00	0	0.08	0	−0.08	−100
*lacc3*	0.42	0.27	0.69	0.20	0.16	−0.49	−71	−0.53	−76	0.03	20
*lacc4*	0.07	0.05	0.12	0.05	0.03	−0.07	−56	−0.09	−73	0.02	62
*lacc5*	0.79	0.82	1.61	1.16	0.43	−0.45	−28	−1.18	−74	0.73	172
*lacc6*	1.12	0.19	1.31	1.36	1.25	0.05	4	−0.06	−5	0.11	9
*lacc7*	0.03	0.01	0.03	0.01	0.02β	−0.02	−70	−0.02	−51	−0.01	−40
*lacc8*	0.02	0.00	0.02	0.00	0.00	−0.01	−87	−0.02	−100	0.00	0
*lacc9*	0.06	0.03	0.10	0.02	0.02	−0.08	−82	−0.07	−78	0.00	−20
*lacc10*	0.14	0.10	0.24	0.19	0.04β	−0.05	−19	−0.20	−85	0.16	446
*lacc11*	0.08	0.02	0.10	0.05	0.07	−0.05	−49	−0.03	−27	−0.02	−30
*lacc12*	0.34	0.06	0.40	0.15	0.11	−0.24	−61	−0.29	−73	0.05	45

a Mean relative expression along the whole culture cycle (units relative to reference index).

b This model considers the sum of the two monokaryotic strains.

α Means that the relative quantity of this dikaryon is significantly different to mk61 and mk63 (p<0.01).

β Means that the relative quantity of dikaryon N001 is significantly different to dikaryon 61×63 (p<0.01).

**Table 6:** Genetic effect in LSC laccase transcription^a^.

			Column 1	Column 2	Column 3	Deviation (2–1)		Deviation (3–1)		Deviation (2–3)	
Gene	mk61	mk63	Additive model^b^	61×63	N001 model	Units	%	Units	%	Units	%
*lacc1*	0.07	0.05	0.12	0.09	0.03β	−0.03	−26	−0.09	−77	0.06	219
*lacc2*	0	0	0	0	0.54^αβ^	0	0	0.54	0	−0.54	−100
*lacc3*	0.26	0.17	0.43	0.27	0.12	−0.16	−38	−0.31	−73	0.15	125
*lacc4*	0.15	0.11	0.26	0.06	0.08	−0.2	−77	−0.18	−71	−0.02	−23
*lacc5*	0.43	0.5	0.94	0.84	1.09α	−0.1	−11	0.15	16	−0.25	−23
*lacc6*	0.03	0.05	0.07	0.33α	0.83^αβ^	0.26	347	0.76	1020	−0.50	−60
*lacc7*	0.03	0.03	0.06	0.05	0.02β	−0.02	−27	−0.05	−76	0.03	198
*lacc8*	0.08	0	0.08	0	0	−0.08	−100	−0.08	−100	0	−100
*lacc9*	0.02	0	0.02	0.06α	0.12α	0.04	193	0.1	505	−0.06	−52
*lacc10*	0.09	0.06	0.15	0.97α	2.57^αβ^	0.82	557	2.42	1639	−1.60	−62
*lacc11*	0.05	0.01	0.06	0.03	0.1β	−0.03	−50	0.05	77	−0.07	−72
*lacc12*	0.09	0.02	0.11	0.03	0.26	−0.07	−68	0.15	138	−0.22	−87

a Mean relative expression along the whole culture cycle (units relative to reference index).

b This model considers the sum of the two monokaryotic strains.

α Means that the relative quantity of this dikaryon is significantly different to mk61 and mk63 (p<0.01).

β Means that the relative quantity of dikaryon N001 is significantly different to dikaryon 61×63 (p<0.01).

**Figure 4:**
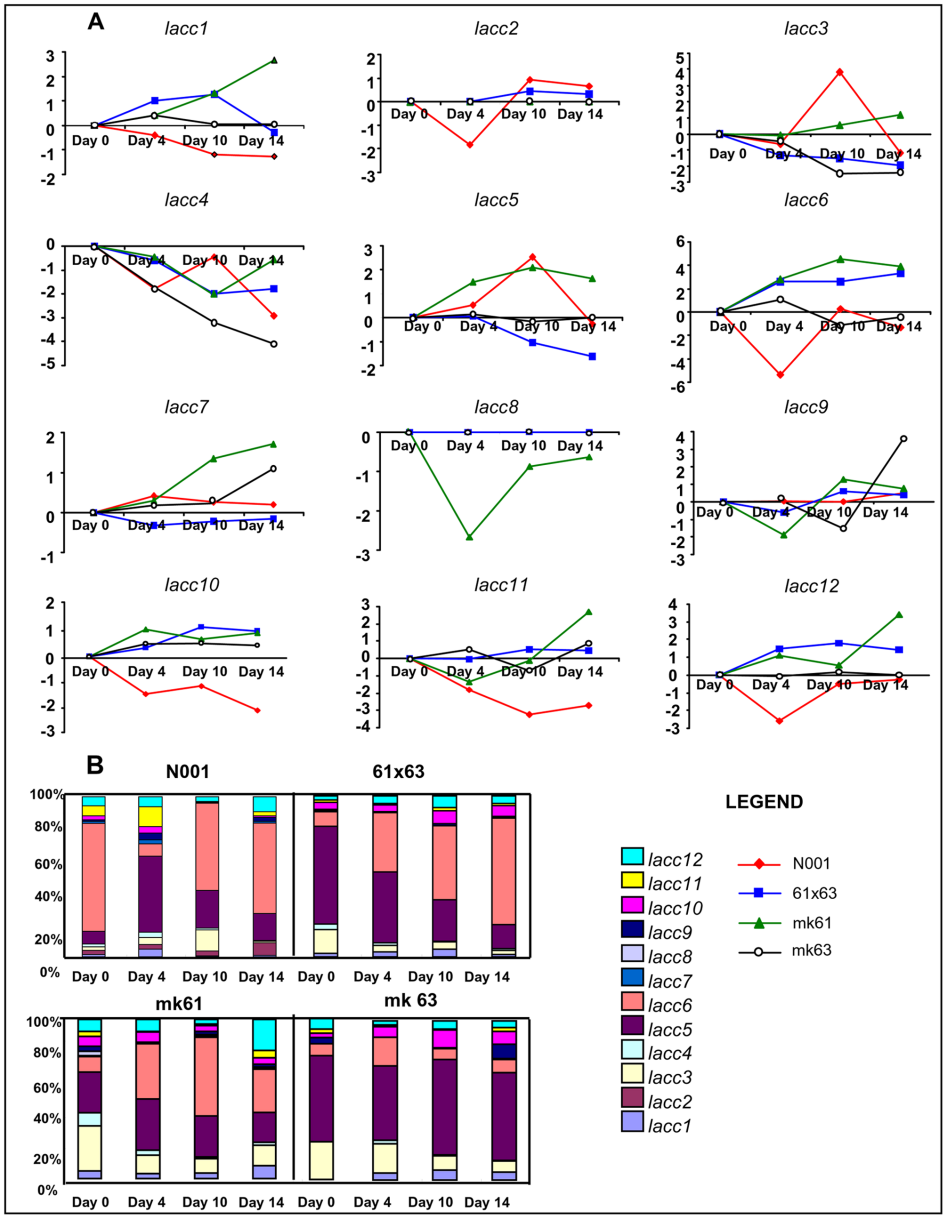
Time course transcriptional profile of *lacc* genes in GSC. The transcript levels were expressed as fold changes (FC) compared to the expression on day 0 (A), and in relative quantities (RQ), which was expressed as a percentage (B). Gene expression ratios with standard errors of the mean are shown in Tables S6 to S9 in File S1.

The mean relative quantities of the different *lacc* genes along the culture cycle in GSC varied from the lack of *lacc2* expression in mk61, mk63 and 61×63, and lack of *lacc8* expression in mk63, 61×63 and N001, to 1.36 relative units in *lacc6* expression in 61×63 (Table 5). Most of the laccase gene expression corresponded to *lacc5* and *lacc6* and to a lower extent, *lacc3, lacc10* and *lacc12* (Figure 4B, Table 5). In general, the transcript levels produced by most of the laccase genes in strains 61×63 and N001 were downregulated in comparison to an additive model, which represented the sum of the relative quantities corresponding to each monokaryon (Table 5). Nevertheless, the results obtained for *lacc6* expression in the dikaryotic strains 61×63 and N001 fitted to what was expected under an additive gene model. Similarly, the detected *lacc5* (a ferroxidase and not a true laccase) and *lacc10* transcripts could also match to what was expected for an additive model in 61×63, but not for N001. In this strain, a clear downregulation was observed in *lacc*5 (74%) and *lacc10* (85%).

**Figure 5:**
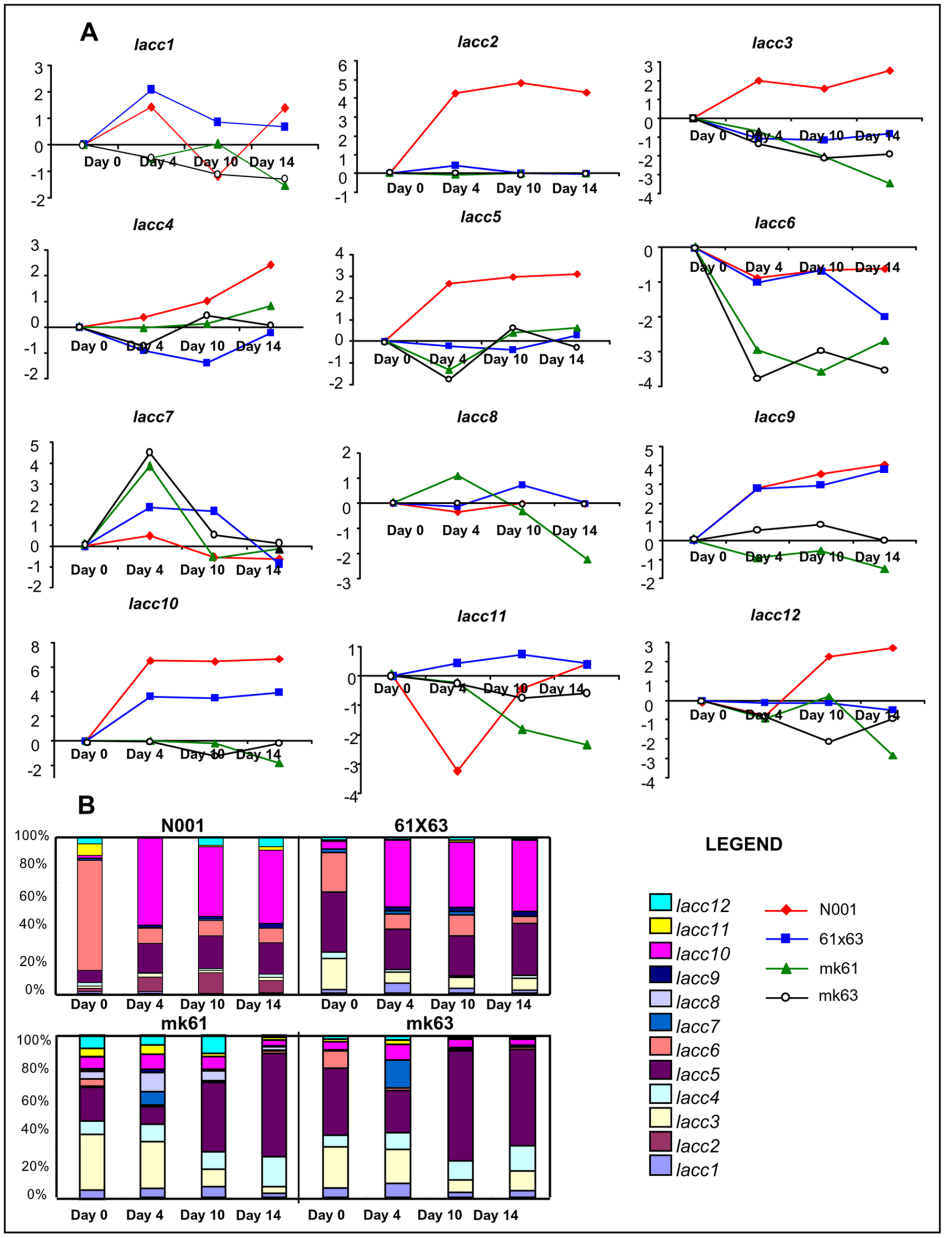
Time course transcriptional profile of *lacc* genes in LSC. Transcript levels were expressed as fold changes (FC) compared to the expression on day 0 (A), and in relative quantities (RQ), which was expressed as a percentage (B). Gene expression ratios with standard errors of the mean are shown in Tables S6 to S9 in File S1.

The mean relative quantities of the different *lacc* genes along the culture cycle in LSC varied from the lack of expression (*lacc2* in mk61, mk63 and 61×63; and *lacc8* in mk63, 61×63 and N001 in the GSC cultures) to 2.57 relative units (*lacc10* in N001) (Table 6). In the LSC cultures, the expression of *lacc2* and *lacc8* showed a similar profile to that observed in the GSC cultures. Most of the laccase gene expression corresponded to *lacc3, lacc5*, *lacc6* and *lacc10* (Table 6, Figure 5B). Considering the monokaryotic or dikaryotic condition of the strain, very significant differences were observed in gene expression. The expression of *lacc2*, *lacc6* and *lacc10* was observed almost exclusively in dikaryons (in the case of *lacc2*, it was expressed only in N001). In N001, the four major transcripts (*lacc2, lacc5*, *lacc6* and *lacc10*) accounted for 87.5% of the total laccase transcripts along the entire cycle; and the combined expression of *lacc5*, *lacc6*, *lacc10* accounted for 79% of the *lacc* transcripts detected in 61×63. In the monokaryotic strains, *lacc3*, *lacc4* and *lacc5* were the predominant transcripts, representing 65% and 78% of total transcripts amount in mk61 and mk63, respectively. Interestingly, the relative quantities of *lacc6*, *lacc9* and *lacc10* in dikaryotic strains 61×63 and N001 were much higher than those expected under an additive model (Table 6), suggesting a clear overdominance effect under this culture condition. Importantly, the expression of *lacc*5 was also higher than what was expected under the additive model, but only in the N001 strain.

Taking together, these data provides a profile of the laccase gene expression during the culture cycle in GSC and LSC. In the GSC cultures (Figure 4B), the expression of *lacc5* and *lacc6* were predominant in all strains, and showed a lower but constitutive presence of *lacc3* and *lacc12* transcripts. A similar study in LSC cultures revealed a predominance of *lacc10* at the expense of *lacc6* in dikaryons, a behaviour that was not observed in monokaryons.

#### (ii) Gene expression profiling using the FC approach and multivariate analysis

**Figure 6:**
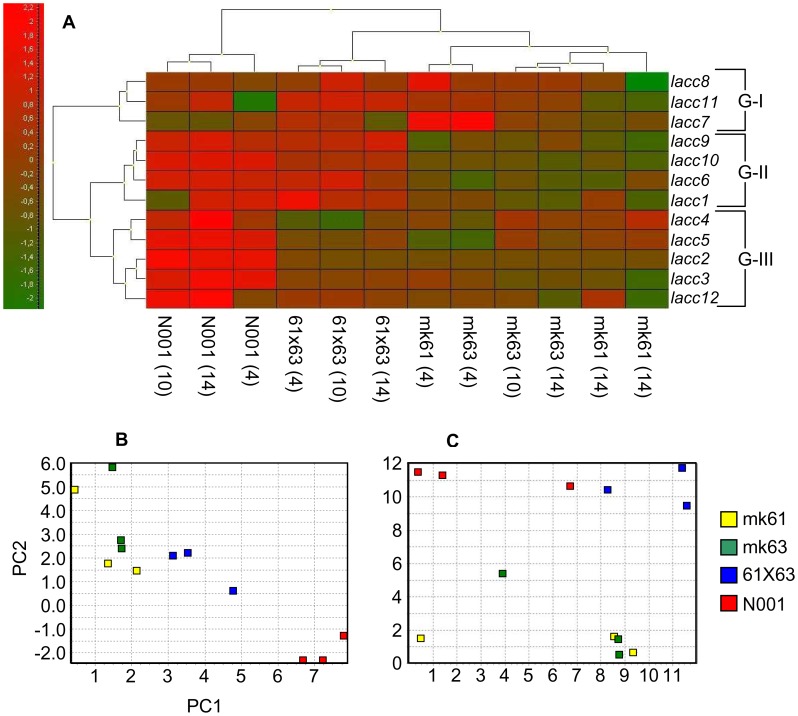
Multivariate analysis of expression profiles. Cluster analysis of genes and samples using a heat map (A), principal component analysis (B) and Kohonen self-organising map (C). Sample loadings of the two principal components are shown in Table S10 in File S1.

The expression of each gene was calculated in relation to day 0, and expressed as the fold change (FC, Materials and Methods, equation 2). This approach reflects the profile of each gene along the culture cycle (Figures 4A and 5A). Although the FC values could not be quantitatively compared among the strains (the reference condition demonstrates different transcription levels in each strain), different expression profiles can be revealed using multivariate analysis (Figure 6).

In GSC cultures (Figure 4A), there was not a clear pattern of variation during the culture cycle. However, in LSC cultures (Figure 5A), the expression profile in dikaryon N001 was clearly different from that of the other strains for *lacc2*, *lacc3*, lacc4, *lacc5*, and *lacc12*, which were upregulated. When LSC cultures of monokaryons and dikaryons were compared, the expression profiles of *lacc6*, *lacc9* and *lacc10* were different. In particular, a reduction in the relative expression of *lacc6* compared to day 0 was observed in the four strains; however, the reduction was much higher in monokaryons mk61 and mk63 compared to dikaryons N001 and 61×63. Similarly, there was an increase in the expression of *lacc9* and *lacc10* in the dikaryons, whereas it remained constant in the monokaryons.

Multivariate exploratory analyses performed with auto-scaled data of LSC cultures enabled the comparison and grouping of *lacc* genes and strains/day of culture (i.e., N001 - day 4) according to the similarity of their expression profiles in a medium where laccases were induced. Regarding gene grouping, three gene groups were obtained using hierarchical clustering (Figure 6A): Group I consisted of *lacc7*, *lacc8*, and *lacc11*; Group II consisted of *lacc1*, *lacc6*, *lacc9* and *lacc10* and Group III consisted of *lacc2*, *lacc3*, *lacc4*, *lacc5* and *lacc12*. Group I genes displayed erratic over- and under-expression in monokaryons and dikaryons, whereas group II genes were overexpressed in dikaryons compared to monokaryons. Furthermore, genes in group III were overexpressed only in the fully heterozygous control strain N001. Regarding sample clustering, the samples obtained from the control strain N001 formed a separate group. The samples from dikaryon 61×63 were separately grouped from its parental monokaryotic strains, and the samples for four day cultures of monokaryons mk61 and mk63 were grouped independently from the samples obtained at later culture times in these two strains.

Principal component analysis (PCA) was performed using mean-centered data. The principal components (PC1+ PC2) accounted for 75.6% of the total variation (Figure 6B), resulting from a 12-dimensional data set (each sample contained the measurements of 12 genes). PC1 differentiated the same sample groups compared to hierarchical clustering, while PC2 separated the N001 (negative values) samples from the samples obtained from the rest of the strains (positive values). PC1 was positively correlated with the expression of *lacc2*, *lacc5*, *lacc6* and *lacc10*. The Kohonen self-organising map (Figure 6C) clearly distinguished between monokaryons (I) and dikaryons (II) according to their differential expression profiles.

## Discussion

### Genetic Control of Growth Rate in Monokaryons and Dikaryons

The genetic bases of growth rate have been extensively studied in cultivated mushrooms due to the importance of this trait in agro industry and biotechnology [22], [23]. However, the effect of monokaryotic and dikaryotic conditions on growth rate has not yet been completely understood [24]–[26]. The current body of literature related to the genetic control of monokaryotic and dikaryotic growth rate in basidiomycetes is limited. In previous studies, we determined the presence of QTLs controlling growth rate in monokaryons and dikaryons cultivated on glucose (MEA) or lignocelullosic (straw) media in *P. ostreratus*. In addition, we mapped these QTLs to chromosomes I, IV and VIII, and determined the extent of the growth rate variation accounted for in monokaryons and dikaryons [22].

In this study, we selected monokaryotic strains that were isogenic for chromosome VIII that ferried identical growth rate QTL alleles (*Qmgre2*, R^2^ = 20.27%, and *Qmgrs2* R^2^ = 11.31%) at this chromosome. However, these strains showed growth rate segregation (Table 1) that could be explained by differences in the growth rate of the QTLs mapped to chromosomes I and/or IV, or in other genomic regions interacting with the QTL mapping to chromosome VIII (intranuclear interactions).

To study the effect of the monokaryotic/dikaryotic condition on growth rate, we generated dikaryotic strains by mating compatible monokaryons, and observed that although these strains had growth rate performances that were significantly higher than those observed for their monokaryotic parentals (Table 1), none of the strains reached the growth rate expected under a theoretical additive gene model, which was defined as the sum of the monokaryotic components. These results suggested that other constraints could affect the growth rate trait in dikaryons, because the growth rate depends, among other factors, on the interactions of each nucleus with an identical cytoplasm (G_1_E)+(G_2_E), the cross-talks between both monokaryotic nuclei (G_1_G_2_), and the interactions of the interacting nuclei with the cytoplasm (G_1_G_2_)E. Thus, the results obtained indicated that multiple nucleus/nuclei/cytoplasmic interactions can modulate the growth rate in a dikaryon. In that sense, James *et al.* [28] reported that factors such as dominance and epistasis were involved in the heterokaryotic growth rate observed in *Heretobasidion parviporum.* In the case of of *Schizophyllum commune,* several dominant mutations resulting from a co-adaptive process between the haploid genomes in an asexual dikaryon have been described [27], which accounted for a higher growth rate.

### Genetic Control of Extracellular Laccase Activity in GSC and LSC Cultures

The *P. ostreatus* genome encodes for 12 laccase genes mapped to chromosomes IV (*lacc3*), VI (cluster described below and *lacc7*), VII (*lacc8*), VIII (*lacc2*) and XI (*lacc5* and *lacc12*). Importantly, the cluster formed by six laccase genes mapped to the subtelomeric region of chromosome VI (*lacc1, lacc4, lacc6, lacc9, lacc10, lacc11)* spans 82 kbp of the genomic sequence. Lacc10 is the most abundant isozyme released into the medium in submerged and solid state cultures [29], and Lacc2 and Lacc10 are the most sensitive when induced by wheat straw-derived phenolic compounds [13]. Thus, the laccase activity released by the strains analysed was much lower in GSC than in LSC (Figure 2). These findings supported the requirement of inducers for efficient laccase activity production. In LSC cultures, the phenolic inducers present in the wheat straw were released into the medium and promoted strong increases in laccase activities in all the tested strains except for mk61 and mk63. These increases were always higher in dikaryons compared to their parental monokaryons, and coincident with transcriptional increments in *lacc9* and *lacc10* (Table S5 in File S1), suggesting that dikaryons were more sensitive than monokaryons to the presence of inducers. Increases in laccase activity in dikaryons are compatible with additive and overdominance effects. These effects are genotype-specific and dependent on culture media, suggesting a tight interaction between genotypes and the environment. The activity profile observed in strain 61×63 and its parental monokaryons mk61 and mk63 cultured in LSC (Figure 2) offered an interesting scenario for further analysis because laccase activities were absent in the monokaryons, but present in the dikaryon, with a homozygous genotype for *lacc2* and *lacc10* (Table S4 in File S1). Thus, we performed a transcriptional analysis of the laccase genes, which could provide clues to uncover the underlying mechanisms of this behaviour as well as the effect of different allelic combinations in laccase gene expression.

### Transcriptional Analysis of *lacc* Genes

Transcription of the laccase family has been quantified using two approaches: Transcriptional units relative to a reference index (RQ, equation 1, Materials and Methods), and fold changes relative to a reference condition (FC, equation 2). The RQ estimated transcript quantities in arbitrary units, and the FC compared the expression profile trends among the strains. The stability of the reference index (Figure 3, Figure S2 in File S2) and the calibration with amplification efficiency made it possible to accurately compare the mean transcript abundance between genes and strains. The transcriptional analysis performed using the RQ approach revealed that the overdominance effect observed in the Lac activity released by dikaryons in lignocellulose-based LSC cultures fitted well with the non-additive changes in the expression of *lacc6, lacc9* and *lacc10.* In relation to the expression pattern of *lacc2*, the high correspondence between the heterozygous genotype of this locus (Table S4 in File S1) and its phenotype (mean relative quantity, Table 6) should be stressed. This correspondence was observed in N001, but not in the full-sib derived dikaryon 61×63 (homozygote, see Table 2) and could explain the higher Lac activities detected in the strain N001. In GSC, *lacc5* and *lacc6* were the most expressed genes. As previously indicated, the Lacc5 isozyme has been proposed to not be a true laccase. It was originally predicted as a laccase, although more recently it has been classified as a Fet3-type fungal ferroxidase [31], a protein involved in high-affinity iron uptake that is transcriptionally repressed by iron in *P. chrysosporium* and in *Saccharomyces cerevisiae* [32], [33]. Lacc6 is a well-defined laccase that has been suggested to be inefficiently secreted, to have an intracellular role, and to be degraded by specific proteases [29]. The increases in *lacc6* expression in dikaryons during the culture cycle suggest the importance of this enzyme while the fungus is active in biomass production.

Analyses using the second approach (fold changes, FC) revealed an apparent erratic behaviour (Figures 4A and 5A), suggesting that the expression patterns may be strongly affected by strain genotype and culture medium. Nevertheless, the combination of this approach with the multivariate analysis yielded some interesting results. For example, in group II, *lacc1*, *lacc6*, *lacc9* and *lacc10* (Figure 6) were upregulated in dikaryons and downregulated in monokaryons cultivated in LSC conditions. These four genes formed a cluster mapped to the chromosome VI subtelomeric region. This clustering suggested that upregulation of the entire gene cluster in dikaryons was the result of a co-regulatory effect. The cluster also contained two other gene models (*lacc4* and *lacc11*). Whereas *lacc4* transcripts appeared to be upregulated in the fully heterozygous N001 strain and downregulated in 61×63, a more erratic expression pattern was observed in *lacc11*. Finally, *lacc2* was upregulated in the heterozygous strain N001, although it was not a member of the gene cluster. This gene is not catalogued as a *sensu stricto* laccase [31] and encodes an unusual enzyme among fungi because it acts as a heterodimer with a small subunit [30], [34].

A multigene family is a group of genes with a common descent that have similar functions and DNA sequences. Small gene families are subjected to a mixed birth and death evolutionary process with strong purifying selection. The laccase genes belong to the multicooper oxidase gene family, which contains five subfamilies. Ten *P. ostreatus* laccase genes belong to the *sensu stricto* subfamily I, *lacc2* to subfamily II, and *lacc5* is a fungal fet-3 ferroxidase [31]. The laccase gene family should not escape the mixed birth and death evolutionary process previously described because inverted gene sequences participating in recombination and gene conversion processes have been observed, as it has been previously described for other gene families such as heat shock proteins and amylase genes. These inverted gene sequences are particularly relevant in the laccase gene cluster found in *P. ostreatus* chromosome VI, where *lacc1*–*lacc11* and *lacc9–lacc4* formed two pairs of inverted genes flanked by *lacc6* and *lacc10*, respectively, which were themselves also inverted. In addition, *lacc9* appeared to be a duplication of *lacc10* because they share the highest sequence similarity among the *lacc* genes, and their expression profiles were very similar, although *lacc9* expression was much lower than that of *lacc10*. Furthermore, these results suggested that the gene organisation and evolutionary process mediated by sequence inversions and gene conversions could offer a special method for the co-regulation of gene expression within the cluster.

## Conclusions

The study of growth rate, laccase activity release and laccase gene transcription performed on *P. ostreatus* strains indicated that (i) monokaryons and dikaryons displayed differential behaviour in growth rate and RBBR-decolorizing ability, (ii) dikaryons displaying good performance in extracellular laccase activity in glucose-based media demonstrated worse behaviour in lignocellulose media and vice-versa, (iii) *lacc6*, *lacc2*, and *lacc10* expression was divergent in GSC and LSC, and their expression was highly correlated with the extracellular laccase activity detected, (iv) *lacc* genes have been classified into three clusters/groups according to their expression profiles in lignocellulosic media, (v) the differences in monokaryon and dikaryon extracellular activities could be explained by non-additive increases in the expression of *lacc2*, *lacc6* and *lacc10,* and (vi) the growth rate, laccase activity and gene expression displayed a non-additive genomic effect in dikaryons compared to their parental monokaryons. The incubation times used in this study cover the exponential, linear and stationary growth phases of *P. ostreatus* in liquid cultures [35]. Thus, our results are applicable for cultures in the framework of enzyme production under laboratory conditions. Nevertheless, the culture periods used reflect a very small part of a complete individual life cycle, in which *P. ostreatus* requires longer periods to cause wood decay. Thus, our results might not be extended to such natural conditions.

## Supporting Information

File S1 **File containing supplemental Tables S1–S10.**
(DOC)

File S2 **File containing Figures S1 and S2. Figure S1:** Remazol brilliant Blue R decolorization scale. **Figure S2:** Mean Cp values and standard deviations of the reference index used for qPCR data normalization in the four strains of this study cultured on GSC and LSC.
(DOC)
