# Supplementary material for: Non-Additive Transcriptional Profiles Underlie Dikaryotic Superiority in Pleurotus ostreatus Laccase Activity
Source: PLoS One. 2013 Sep 5;8(9):e73282. doi: 10.1371/journal.pone.0073282 (PMC3764117; doi:10.1371/journal.pone.0073282)
Supplement: File S1 — File containing supplemental Tables S1–S10. (DOC) [file pone.0073282.s001.doc]

**Table S1.** Primers used for allele type identification.

|  | Gene ID | |  |  | Amplicon |
| --- | --- | --- | --- | --- | --- |
| Gene | PC15 v2.0 | PC9 v1.0 |  | Primer sequence | length (bp) |
| *lacc1* | 1043420 | 90578 | Fw | CGACAGCACGCTTATCAATG | 994 |
|  |  |  | Rv | CTTTTTCCTCGTCGGTGAGT |  |
| *lacc4* | 1077328 | 65894 | Fw | ACATTGGCACGGTCTCTACC | 1022 |
|  |  |  | Rv | GACCGTGAAGATGGAAAGGA |  |
| *lacc6* | 1113032 | 81104 | Fw | CATTGATCAAGGGCAAGACC | 1068 |
|  |  |  | Rv | ATACGAGGGCAGGCATAGTG |  |
| *lacc11* | 1043488 | 90573 | Fw | GCCTCTGATCGCAGGAAATA | 1070 |
|  |  |  | Rv | CCAGCGGTGTCAAGGTTAAT |  |

**Table S2.** Laccase activities in GSC and LSC

| **Strain** | **Days** | **GSC** | **SD** | **LSC** | **SD** |
| --- | --- | --- | --- | --- | --- |
| mk36 | 0 | 0.00 | 0.00 | 0.00 | 0.00 |
| mk36 | 4 | 0.13 | 0.05 | 7.53 | 3.09 |
| mk36 | 10 | 0.78 | 0.24 | 20.88 | 7.38 |
| mk36 | 14 | 2.03 | 1.19 | 20.67 | 5.44 |
| mk61 | 0 | 0.00 | 0.00 | 0.00 | 0.00 |
| mk61 | 4 | 0.98 | 0.17 | 0.18 | 0.10 |
| mk61 | 10 | 5.20 | 1.77 | 0.00 | 0.00 |
| mk61 | 14 | 2.08 | 1.40 | 0.18 | 0.04 |
| mk63 | 0 | 0.00 | 0.00 | 0.00 | 0.00 |
| mk63 | 4 | 0.23 | 0.05 | 0.24 | 0.10 |
| mk63 | 10 | 0.65 | 0.06 | 0.55 | 0.22 |
| mk63 | 14 | 0.30 | 0.08 | 0.70 | 0.22 |
| mk67 | 0 | 0.00 | 0.00 | 0.00 | 0.00 |
| mk67 | 4 | 0.33 | 0.05 | 13.00 | 4.28 |
| mk67 | 10 | 2.30 | 0.46 | 73.38 | 16.25 |
| mk67 | 14 | 7.08 | 4.73 | 78.00 | 15.41 |
| mk69 | 0 | 0.00 | 0.00 | 0.00 | 0.00 |
| mk69 | 4 | 0.23 | 0.10 | 6.26 | 1.81 |
| mk69 | 10 | 0.28 | 0.05 | 58.95 | 33.97 |
| mk69 | 14 | 1.20 | 0.06 | 82.50 | 15.08 |
| mk93 | 0 | 0.00 | 0.00 | 0.00 | 0.00 |
| mk93 | 4 | 0.65 | 0.31 | 9.50 | 2.60 |
| mk93 | 10 | 2.08 | 1.50 | 22.88 | 10.13 |
| mk93 | 14 | 7.10 | 3.39 | 21.75 | 3.49 |
| N001 | 0 | 0.00 | 0.00 | 0.00 | 0.00 |
| N001 | 4 | 0.80 | 0.10 | 8.30 | 2.35 |
| N001 | 10 | 1.77 | 0.31 | 90.15 | 12.53 |
| N001 | 14 | 4.47 | 1.11 | 93.50 | 11.65 |
| 36x69 | 0 | 0.00 | 0.00 | 0.00 | 0.00 |
| 36x69 | 4 | 1.13 | 0.38 | 2.67 | 1.01 |
| 36x69 | 10 | 5.33 | 0.95 | 106.83 | 18.69 |
| 36x69 | 14 | 5.20 | 0.44 | 77.63 | 20.02 |
| 61x63 | 0 | 0.00 | 0.00 | 0.00 | 0.00 |
| 61x63 | 4 | 15.20 | 3.40 | 0.43 | 0.33 |
| 61x63 | 10 | 13.23 | 1.33 | 14.08 | 3.83 |
| 61x63 | 14 | 6.90 | 2.09 | 38.50 | 11.05 |
| 67x69 | 0 | 0.00 | 0.00 | 0.00 | 0.00 |
| 67x69 | 4 | 1.20 | 0.20 | 17.70 | 2.73 |
| 67x69 | 10 | 2.53 | 0.12 | 75.75 | 19.67 |
| 67x69 | 14 | 1.83 | 0.86 | 187.75 | 35.36 |
| 93x69 | 0 | 0.00 | 0.00 | 0.00 | 0.00 |
| 93x69 | 4 | 2.03 | 0.90 | 27.98 | 6.14 |
| 93x69 | 10 | 3.37 | 0.45 | 207.25 | 47.40 |
| 93x69 | 14 | 3.17 | 1.16 | 300.00 | 18.14 |

**Table S3.** Statistical analysis (Tukey’s test) of laccase production of N001, monokaryons and new dikaryon strains cultivated in GSC (A) and LSC (B).

**A)**

| **Strain** | **Mean** | **Group I** | **Group II** |
| --- | --- | --- | --- |
| mk63 | 0,39 | A |  |
| mk69 | 0,57 | A |  |
| mk36 | 0,98 | A |  |
| 67x69 | 1,85 | A |  |
| N001 | 2,35 | A |  |
| mk61 | 2,75 | A |  |
| 93x69 | 2,86 | A |  |
| mk67 | 3,24 | A |  |
| mk93 | 3,28 | A |  |
| 36x69 | 3,89 | A |  |
| 61x63 | 11,78 |  | B |
| **p value** |  | 0,739 | 1 |

Groups with the same letter display p values > 0.05.

B)

| **Strain** | **Mean** | **Group I** | **Group II** | **Group III** | **Group IV** |
| --- | --- | --- | --- | --- | --- |
| mk61 | 0.125 | A |  |  |  |
| mk63 | 0.508 | A |  |  |  |
| mk36 | 16.3 | A |  |  |  |
| 61x63 | 17.666 | A |  |  |  |
| mk93 | 18.041 | A |  |  |  |
| mk69 | 49.025 |  | B |  |  |
| mk67 | 54.791 |  | B |  |  |
| 36x69 | 62.402 |  | B |  |  |
| N001 | 63.983 |  | B |  |  |
| 67x69 | 93.733 |  |  | C |  |
| 93x69 | 179.366 |  |  |  | D |
| **p value** |  | 0,291 | 0,561 | 1 | 1 |

Groups with the same letter display p values > 0.05.

**Table S4.** Genotype of the *lacc* loci in the analyzed strains

| **Gene** | **N001** | **mk61** | **mk63** | **61x63** | **93x69** | **67x69** | **36x69** |
| --- | --- | --- | --- | --- | --- | --- | --- |
| *lacc1* | Aa | A | a | Aa |  |  |  |
| *lacc2* | Aa | A | A | AA | AA | AA | AA |
| *lacc3* | Aa | A | a | Aa |  |  |  |
| *lacc4* | Aa | a | a | aa |  |  |  |
| *lacc5* | Aa | A | a | Aa |  |  |  |
| *lacc6* | Aa | A | a | Aa |  |  |  |
| *lacc7* | Aa | A | a | Aa |  |  |  |
| *lacc8* | - a | a | - | - a |  |  |  |
| *lacc9* | Aa | A | a | Aa |  |  |  |
| *lacc10* | Aa | a | a | aa | Aa | AA | Aa |
| *lacc11* | Aa | a | a | aa |  |  |  |
| *lacc12* | Aa | A | a | Aa |  |  |  |

A = PC9 allele type

a = PC15 allele type

**Table S5.** Statistical analysis of relative quantities detected in LSC condition *versus* GSC condition.

|  | **LSC vs GSC** | | | | | | | |
| --- | --- | --- | --- | --- | --- | --- | --- | --- |
| **Gene** | **N001** | | **61** | | **63** | | **61x63** | |
|  | **UP** | **DOWN** | **UP** | **DOWN** | **UP** | **DOWN** | **UP** | **DOWN** |
| *lacc1* |  |  |  |  |  |  |  |  |
| *lacc2* | p<0.001 |  |  |  |  |  |  |  |
| *lacc3* |  |  |  |  |  |  |  |  |
| *lacc4* | p<0.001 |  | p<0.001 |  | 0,001 |  |  |  |
| *lacc5* | p<0.001 |  |  |  |  |  |  |  |
| *lacc6* |  |  |  | p<0.001 |  | p<0.001 |  |  |
| *lacc7* |  |  |  |  |  |  |  |  |
| *lacc9* | p<0.001 |  |  |  |  |  | p<0.001 |  |
| *lacc10* | p<0.001 |  |  |  |  |  | p<0.001 |  |
| *lacc11* |  |  |  |  |  |  |  |  |
| *lacc12* |  |  |  |  |  | 0,009 |  | p<0.001 |

Differences were considered statistically significant when these two conditions met:

p < 0.01, and the transcripts in one of the conditions tested doubled the other.

**Table S6.** Gene expression ratios of lacc genes obtained in N001 expressed in linear scale, according to formula 2. Standard errors of the mean (SEM) are included.

| N001 |  |  |  |  |  |
| --- | --- | --- | --- | --- | --- |
|  |  | GSC | | LSC | |
| Target | Time (days) | Ratio | SEM | Ratio | SEM |
| *lacc1* | 0 | 1.00 | 0.35 | 1.00 | 0.23 |
| *lacc1* | 4 | 0.76 | 0.12 | 2.66 | 0.44 |
| *lacc1* | 10 | 0.44 | 0.12 | 0.44 | 0.03 |
| *lacc1* | 14 | 0.41 | 0.16 | 2.64 | 0.58 |
| *lacc2* | 0 | 1.00 | 0.15 | 1.00 | 0.28 |
| *lacc2* | 4 | 0.28 | 0.04 | 19.44 | 5.17 |
| *lacc2* | 10 | 1.90 | 0.50 | 28.54 | 2.25 |
| *lacc2* | 14 | 1.56 | 0.22 | 19.86 | 2.64 |
| *lacc3* | 0 | 1.00 | 0.16 | 1.00 | 0.11 |
| *lacc3* | 4 | 0.64 | 0.06 | 4.03 | 0.55 |
| *lacc3* | 10 | 14.31 | 1.57 | 2.99 | 0.72 |
| *lacc3* | 14 | 0.44 | 0.09 | 5.76 | 0.84 |
| *lacc4* | 0 | 1.00 | 0.41 | 1.00 | 0.21 |
| *lacc4* | 4 | 0.29 | 0.21 | 1.30 | 0.14 |
| *lacc4* | 10 | 0.73 | 0.08 | 2.03 | 0.55 |
| *lacc4* | 14 | 0.13 | 0.04 | 5.40 | 0.68 |
| *lacc5* | 0 | 1.00 | 0.19 | 1.00 | 0.09 |
| *lacc5* | 4 | 1.44 | 0.08 | 6.35 | 0.42 |
| *lacc5* | 10 | 5.82 | 0.42 | 7.82 | 0.42 |
| *lacc5* | 14 | 0.84 | 0.08 | 8.60 | 0.59 |
| *lacc6* | 0 | 1.00 | 0.21 | 1.00 | 0.09 |
| *lacc6* | 4 | 0.02 | 0.00 | 0.54 | 0.03 |
| *lacc6* | 10 | 1.22 | 0.09 | 0.63 | 0.09 |
| *lacc6* | 14 | 0.39 | 0.03 | 0.65 | 0.08 |
| *lacc7* | 0 | 1.00 | 0.36 | 1.00 | 0.17 |
| *lacc7* | 4 | 1.34 | 0.41 | 1.42 | 0.36 |
| *lacc7* | 10 | 1.20 | 0.36 | 0.68 | 0.08 |
| *lacc7* | 14 | 1.15 | 0.18 | 0.65 | 0.04 |
| *lacc8* | 0 | 0.00 | 0.00 | 1.00 | 0.09 |
| *lacc8* | 4 | 0.00 | 0.00 | 0.79 | 0.08 |
| *lacc8* | 10 | 0.00 | 0.00 | 0.00 | 0.00 |
| *lacc8* | 14 | 0.00 | 0.00 | 0.00 | 0.00 |
| *lacc9* | 0 | 1.00 | 0.35 | 1.00 | 0.50 |
| *lacc9* | 4 | 1.02 | 0.06 | 6.92 | 0.62 |
| *lacc9* | 10 | 0.00 | 0.00 | 11.71 | 1.54 |
| *lacc9* | 14 | 1.43 | 0.60 | 16.17 | 1.92 |
| *lacc10* | 0 | 1.00 | 0.12 | 1.00 | 0.27 |
| *lacc10* | 4 | 0.36 | 0.05 | 91.42 | 4.38 |
| *lacc10* | 10 | 0.45 | 0.05 | 86.05 | 5.92 |
| *lacc10* | 14 | 0.23 | 0.13 | 100.05 | 10.30 |
| *lacc11* | 0 | 1.00 | 0.05 | 1.00 | 0.17 |
| *lacc11* | 4 | 0.29 | 0.22 | 0.11 | 0.05 |
| *lacc11* | 10 | 0.11 | 0.02 | 0.75 | 0.12 |
| *lacc11* | 14 | 0.15 | 0.07 | 1.32 | 0.15 |
| *lacc12* | 0 | 1.00 | 0.10 | 1.00 | 0.13 |
| *lacc12* | 4 | 0.17 | 0.10 | 0.57 | 0.12 |
| *lacc12* | 10 | 0.72 | 0.13 | 4.79 | 0.72 |
| *lacc12* | 14 | 0.85 | 0.08 | 6.44 | 0.52 |

**Table S7.** Gene expression ratios of lacc genes obtained in 61X63 expressed in linear scale, according to formula 2. Standard errors of the mean (SEM) are included.

| 61X63 |  |  |  |  |  |
| --- | --- | --- | --- | --- | --- |
|  |  | GSC | | LSC | |
| Target | Time (days) | Ratio | SEM | Ratio | SEM |
| *lacc1* | 0 | 1.00 | 0.07 | 1.00 | 0.50 |
| *lacc1* | 4 | 2.01 | 0.13 | 4.19 | 0.28 |
| *lacc1* | 10 | 2.40 | 0.19 | 1.83 | 0.15 |
| *lacc1* | 14 | 0.82 | 0.10 | 1.60 | 0.23 |
| *lacc2* | 0 | 1.00 | 0.07 | 1.00 | 0.03 |
| *lacc2* | 4 | 0.00 | 0.00 | 1.34 | 0.06 |
| *lacc2* | 10 | 1.35 | 0.08 | 1.00 | 0.04 |
| *lacc2* | 14 | 1.25 | 0.04 | 0.99 | 0.04 |
| *lacc3* | 0 | 1.00 | 0.08 | 1.00 | 0.10 |
| *lacc3* | 4 | 0.40 | 0.05 | 0.47 | 0.05 |
| *lacc3* | 10 | 0.35 | 0.04 | 0.45 | 0.04 |
| *lacc3* | 14 | 0.25 | 0.03 | 0.56 | 0.03 |
| *lacc4* | 0 | 1.00 | 0.07 | 1.00 | 0.33 |
| *lacc4* | 4 | 0.67 | 0.04 | 0.53 | 0.21 |
| *lacc4* | 10 | 0.25 | 0.02 | 0.38 | 0.06 |
| *lacc4* | 14 | 0.29 | 0.11 | 0.86 | 0.08 |
| *lacc5* | 0 | 1.00 | 0.08 | 1.00 | 0.04 |
| *lacc5* | 4 | 1.04 | 0.18 | 0.85 | 0.06 |
| *lacc5* | 10 | 0.48 | 0.07 | 0.76 | 0.06 |
| *lacc5* | 14 | 0.32 | 0.03 | 1.23 | 0.11 |
| *lacc6* | 0 | 1.00 | 0.09 | 1.00 | 0.07 |
| *lacc6* | 4 | 6.12 | 0.54 | 0.49 | 0.02 |
| *lacc6* | 10 | 6.02 | 0.68 | 0.63 | 0.07 |
| *lacc6* | 14 | 10.20 | 0.42 | 0.25 | 0.01 |
| *lacc7* | 0 | 1.00 | 0.20 | 1.00 | 0.86 |
| *lacc7* | 4 | 0.80 | 0.29 | 3.60 | 0.73 |
| *lacc7* | 10 | 0.87 | 0.30 | 3.17 | 0.32 |
| *lacc7* | 14 | 0.90 | 0.20 | 0.55 | 0.16 |
| *lacc8* | 0 | 0.00 | 0.00 | 1.00 | 0.05 |
| *lacc8* | 4 | 0.00 | 0.08 | 0.91 | 0.07 |
| *lacc8* | 10 | 0.00 | 0.17 | 1.66 | 0.27 |
| *lacc8* | 14 | 0.00 | 0.06 | 0.00 | 0.00 |
| *lacc9* | 0 | 1.00 | 0.63 | 1.00 | 0.32 |
| *lacc9* | 4 | 0.66 | 0.12 | 6.83 | 3.89 |
| *lacc9* | 10 | 1.52 | 0.19 | 7.80 | 2.93 |
| *lacc9* | 14 | 1.31 | 0.21 | 13.74 | 3.12 |
| *lacc10* | 0 | 1.00 | 0.07 | 1.00 | 0.21 |
| *lacc10* | 4 | 1.26 | 0.22 | 11.86 | 1.01 |
| *lacc10* | 10 | 2.22 | 0.15 | 11.18 | 1.73 |
| *lacc10* | 14 | 2.01 | 0.20 | 15.06 | 1.99 |
| *lacc11* | 0 | 1.00 | 0.35 | 1.00 | 0.54 |
| *lacc11* | 4 | 0.97 | 0.45 | 1.35 | 0.65 |
| *lacc11* | 10 | 1.44 | 0.68 | 1.66 | 0.29 |
| *lacc11* | 14 | 1.37 | 0.48 | 1.37 | 0.46 |
| *lacc12* | 0 | 1.00 | 0.20 | 1.00 | 0.17 |
| *lacc12* | 4 | 2.84 | 0.34 | 0.92 | 0.29 |
| *lacc12* | 10 | 3.50 | 0.53 | 0.92 | 0.42 |
| *lacc12* | 14 | 2.65 | 0.28 | 0.71 | 0.28 |

**Table S8.** Gene expression ratios of lacc genes obtained in 63 expressed expressed in linear scale, according to formula 2. Standard errors of the mean (SEM) are included.

| **mk63** |  |  |  |  |  |
| --- | --- | --- | --- | --- | --- |
|  |  | **GSC** | | **LSC** | |
| **Target** | **Time (days)** | **Ratio** | **SEM** | **Ratio** | **SEM** |
| *lacc1* | 0 | 1.00 | 0.23 | 1.00 | 0.11 |
| *lacc1* | 4 | 1.34 | 0.29 | 0.71 | 0.15 |
| *lacc1* | 10 | 1.04 | 0.14 | 0.47 | 0.14 |
| *lacc1* | 14 | 1.04 | 0.09 | 0.41 | 0.11 |
| *lacc2* | 0 | 1.00 | 0.07 | 1.00 | 0.07 |
| *lacc2* | 4 | 0 | 0 | 0 | 0 |
| *lacc2* | 10 | 0 | 0 | 0 | 0 |
| *lacc2* | 14 | 0 | 0 | 0 | 0 |
| *lacc3* | 0 | 1.00 | 0.13 | 1.00 | 0.12 |
| *lacc3* | 4 | 0.73 | 0.16 | 0.38 | 0.06 |
| *lacc3* | 10 | 0.18 | 0.03 | 0.23 | 0.10 |
| *lacc3* | 14 | 0.19 | 0.07 | 0.26 | 0.06 |
| *lacc4* | 0 | 1.00 | 0.18 | 1.00 | 0.09 |
| *lacc4* | 4 | 0.30 | 0.12 | 0.61 | 0.12 |
| *lacc4* | 10 | 0.11 | 0.02 | 1.36 | 0.17 |
| *lacc4* | 14 | 0.06 | 0.02 | 1.05 | 0.17 |
| *lacc5* | 0 | 1.00 | 0.21 | 1.00 | 0.18 |
| *lacc5* | 4 | 1.12 | 0.24 | 0.30 | 0.03 |
| *lacc5* | 10 | 0.89 | 0.08 | 1.53 | 0.16 |
| *lacc5* | 14 | 1.01 | 0.08 | 0.82 | 0.09 |
| *lacc6* | 0 | 1.00 | 0.35 | 1.00 | 0.14 |
| *lacc6* | 4 | 2.09 | 0.34 | 0.07 | 0.01 |
| *lacc6* | 10 | 0.45 | 0.04 | 0.13 | 0.02 |
| *lacc6* | 14 | 0.73 | 0.06 | 0.09 | 0.01 |
| *lacc7* | 0 | 1.00 | 0.21 | 1.00 | 0.13 |
| *lacc7* | 4 | 1.14 | 0.22 | 22.58 | 1.74 |
| *lacc7* | 10 | 1.17 | 0.50 | 1.48 | 0.21 |
| *lacc7* | 14 | 2.14 | 0.62 | 1.09 | 0.15 |
| *lacc8* | 0 | 0 | 0 | 0 | 0 |
| *lacc8* | 4 | 0 | 0 | 0 | 0 |
| *lacc8* | 10 | 0 | 0 | 0 | 0 |
| *lacc8* | 14 | 0 | 0 | 0 | 0 |
| *lacc9* | 0 | 1.00 | 0.04 | 1.00 | 0.04 |
| *lacc9* | 4 | 0 | 0 | 1.47 | 0.08 |
| *lacc9* | 10 | 0.34 | 0.02 | 1.81 | 0.22 |
| *lacc9* | 14 | 12.12 | 1.04 | 0 | 0 |
| *lacc10* | 0 | 1.65 | 0.39 | 1.00 | 0.27 |
| *lacc10* | 4 | 1.44 | 0.15 | 0.93 | 0.04 |
| *lacc10* | 10 | 1.42 | 0.10 | 0.42 | 0.04 |
| *lacc10* | 14 | 16.97 | 1.52 | 0.86 | 0.34 |
| *lacc11* | 0 | 1.00 | 0.45 | 1.00 | 0.45 |
| *lacc11* | 4 | 1.45 | 0.22 | 0.85 | 0.41 |
| *lacc11* | 10 | 0.61 | 0.05 | 0.60 | 0.07 |
| *lacc11* | 14 | 1.84 | 1.08 | 0.66 | 0.08 |
| *lacc12* | 0 | 1.00 | 0.13 | 1.00 | 0.44 |
| *lacc12* | 4 | 0.96 | 0.23 | 0.56 | 0.19 |
| *lacc12* | 10 | 1.13 | 0.19 | 0.23 | 0.09 |
| *lacc12* | 14 | 1.00 | 0.44 | 0.51 | 0.11 |

**Table S9.** Gene expression ratios of lacc genes obtained in 61 expressed expressed in linear scale, according to formula 2. Standard errors of the mean (SEM) are included.

| **mk61** | | | | | |
| --- | --- | --- | --- | --- | --- |
|  |  | **GSC** | | **LSC** | |
| **Target** | **Time (days)** | **Ratio** | **SEM** | **Ratio** | **SEM** |
| *lacc1* | 0 | 1.00 | 0.10 | 1.00 | 0.13 |
| *lacc1* | 4 | 1.33 | 0.33 | 0.71 | 0.12 |
| *lacc1* | 10 | 2.48 | 0.20 | 1.04 | 0.17 |
| *lacc1* | 14 | 6.35 | 0.50 | 0.34 | 0.10 |
| *lacc2* | 0 | 0 | 0 | 1.00 | 0.10 |
| *lacc2* | 4 | 0 | 0.17 | 0.94 | 0.11 |
| *lacc2* | 10 | 0 | 0.78 | 0 | 0 |
| *lacc2* | 14 | 0 | 0.67 | 0 | 0 |
| *lacc3* | 0 | 1.00 | 0.07 | 1.00 | 0.11 |
| *lacc3* | 4 | 0.94 | 0.07 | 0.62 | 0.12 |
| *lacc3* | 10 | 1.43 | 0.12 | 0.24 | 0.08 |
| *lacc3* | 14 | 2.29 | 0.30 | 0.09 | 0.03 |
| *lacc4* | 0 | 1.00 | 0.10 | 1.00 | 0.14 |
| *lacc4* | 4 | 0.73 | 0.07 | 0.97 | 0.20 |
| *lacc4* | 10 | 0.24 | 0.07 | 1.10 | 0.26 |
| *lacc4* | 14 | 0.68 | 0.31 | 1.79 | 0.26 |
| *lacc5* | 0 | 1.00 | 0.18 | 1.00 | 0.05 |
| *lacc5* | 4 | 2.83 | 0.61 | 0.39 | 0.05 |
| *lacc5* | 10 | 4.26 | 0.57 | 1.29 | 0.13 |
| *lacc5* | 14 | 3.11 | 0.46 | 1.52 | 0.18 |
| *lacc6* | 0 | 1.00 | 0.15 | 1.00 | 0.17 |
| *lacc6* | 4 | 7.00 | 3.52 | 0.13 | 0.03 |
| *lacc6* | 10 | 23.26 | 9.66 | 0.08 | 0.03 |
| *lacc6* | 14 | 14.77 | 1.31 | 0.15 | 0.04 |
| *lacc7* | 0 | 1.00 | 0.11 | 1.00 | 0.08 |
| *lacc7* | 4 | 1.23 | 0.15 | 14.67 | 0.64 |
| *lacc7* | 10 | 2.56 | 0.45 | 0.66 | 0.07 |
| *lacc7* | 14 | 3.28 | 0.51 | 0.92 | 0.12 |
| *lacc8* | 0 | 1.00 | 0.28 | 1.00 | 0.11 |
| *lacc8* | 4 | 0.16 | 0.06 | 2.13 | 0.13 |
| *lacc8* | 10 | 0.55 | 0.09 | 0.81 | 0.24 |
| *lacc8* | 14 | 0.65 | 0.12 | 0.21 | 0.04 |
| *lacc9* | 0 | 1.00 | 0.18 | 1.00 | 0.32 |
| *lacc9* | 4 | 0.27 | 0.03 | 0.54 | 0.35 |
| *lacc9* | 10 | 2.44 | 1.00 | 0.70 | 0.15 |
| *lacc9* | 14 | 1.70 | 0.85 | 0.36 | 0.18 |
| *lacc10* | 0 | 1.00 | 0.16 | 1.00 | 0.10 |
| *lacc10* | 4 | 2.09 | 0.27 | 0.97 | 0.11 |
| *lacc10* | 10 | 1.60 | 0.74 | 0.85 | 0.24 |
| *lacc10* | 14 | 1.90 | 1.26 | 0.28 | 0.19 |
| *lacc11* | 0 | 1.00 | 0.29 | 1.00 | 0.14 |
| *lacc11* | 4 | 0.39 | 0.19 | 0.85 | 0.10 |
| *lacc11* | 10 | 0.91 | 0.45 | 0.29 | 0.10 |
| *lacc11* | 14 | 6.46 | 1.77 | 0.20 | 0.02 |
| *lacc12* | 0 | 1.00 | 0.15 | 1.00 | 0.11 |
| *lacc12* | 4 | 2.16 | 0.12 | 0.52 | 0.11 |
| *lacc12* | 10 | 1.47 | 0.64 | 1.15 | 0.12 |
| *lacc12* | 14 | 10.70 | 2.45 | 0.14 | 0.06 |

**Table S10.** Sample loadings of the two principal components extracted

|  | PC1 | PC2 |
| --- | --- | --- |
| Day4_mk61 | 0.43 | 4.89 |
| Day10_mk61 | 2.14 | 1.46 |
| Day14_mk61 | 1.37 | 1.78 |
| Day4_mk63 | 1.47 | 5.88 |
| Day10_mk63 | 1.73 | 2.38 |
| Day14_mk63 | 1.71 | 2.73 |
| Day4_N001 | 7.84 | - 1.29 |
| Day10_N001 | 7.25 | - 2.32 |
| Day14_N001 | 6.70 | - 2.34 |
| Day4_61x63 | 3.53 | + 2.22 |
| Day10_61x63 | 3.15 | + 2.13 |
| Day14_61x63 | 4.80 | + 0.60 |
