# Supplementary material for: Non-Additive Transcriptional Profiles Underlie Dikaryotic Superiority in Pleurotus ostreatus Laccase Activity
Source: PLoS One. 2013 Sep 5;8(9):e73282. doi: 10.1371/journal.pone.0073282 (PMC3764117; doi:10.1371/journal.pone.0073282)
Supplement: File S2 — File containing Figures S1 and S2. Figure S1: Remazol brilliant Blue R decolorization scale. Figure S2: Mean Cp values and standard deviations of the reference index used for qPCR data normalization in the four strains of this study cultured on GSC and LSC. (DOC) [file pone.0073282.s002.doc]

**Figure S1.** Remazol brilliant Blue R decolorization scale.


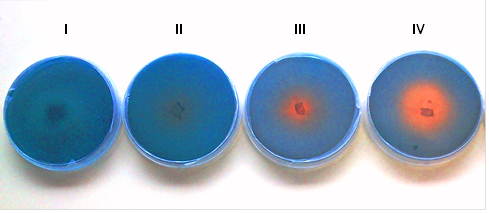


**Figure S2.** Mean Cp values and standard deviations of the reference index used for qPCR data normalization in the four strains of this study cultured on GSC and LSC.

22

23

24

25

26

27

28

29

30

mk61

mk63

61x63

N001

s

-

SSF

SmF

22

23

24

25

26

27

28

29

30

mk61

mk63

61x63

N001

LSC

GSC
